# Intake of Non-steroidal Anti-inflammatory Drugs and the Risk of Prostate Cancer: A Meta-Analysis

**DOI:** 10.3389/fonc.2018.00437

**Published:** 2018-10-23

**Authors:** Zhenhua Shang, Xue Wang, Hao Yan, Bo Cui, Qi Wang, Jiangtao Wu, Xin Cui, Jin Li, Tongwen Ou, Kun Yang

**Affiliations:** ^1^Department of Urology, Xuanwu Hospital Capital Medical University, Beijing, China; ^2^Department of Library, Xuanwu Hospital Capital Medical University, Beijing, China; ^3^Department of Evidence-Based Medicine, Xuanwu Hospital Capital Medical University, Beijing, China

**Keywords:** non-steroidal anti-inflammatory drugs, aspirin, prostate cancer, risk, meta-analysis

## Abstract

**Background:** Epidemiological evidences regarding the association between the use of non-steroidal anti-inflammatory drugs (NSAIDs) and the risk of prostate cancer (PC) is still controversial. Therefore, we conducted a meta-analysis to explore the controversy that exists.

**Methods:** Electronic databases including Medline, EMBASE, Web of Science, Cochrane Library, BIOSIS, Scopus, CBM, CNKI, WANFANG, and CQVIP were used to search for and identify eligible studies published until December 31, 2017. Pooled effect estimates for the relative risk (RR) were computed through fixed-effects or random-effects models as appropriate. Publication bias was evaluated by Egger's and Begg's tests and potential sources of heterogeneity were investigated in subgroup analyses.

**Results:** A total of 43 observational studies were eligible for this meta-analysis. A protective effect was identified for the intake of any NSAIDs on the risk of PC (pooled RR = 0.89, 95% CI = 0.81–0.98). Moreover, the long-term intake of NSAIDs (≥5 years rather than ≥4 years) was associated with reduced PC incidence (pooled RR = 0.882, 95% CI = 0.785–0.991). Aspirin intake was also associated with a 7.0% risk reduction of PC (pooled RR = 0.93, 95% CI = 0.89–0.96). The inverse association became stronger for advanced PC and PC with a Gleason score ≥7 compared to the association with total PC. Interestingly, it was the daily dose (≥1 pill/day) rather than, long-term aspirin intake (≥4 or ≥5 years) that was associated with reduced PC incidence (pooled RR = 0.875, 95% CI = 0.792–0.967). The pooled effects for non-aspirin NSAIDs demonstrated no significantly adverse or beneficial effects on total PC, advanced PC, or PC with Gleason score ≥7, though all pooled RRs were >1.

**Conclusions:** Our findings suggested a protective effect of the intake of any NSAIDs on the risk of PC, especially in those who took the NSAIDs for a long period. Moreover, aspirin intake was also associated with a decreased risk of PC, and there was a dose related association between aspirin intake and the risk of PC, while no significant effects of long-term aspirin intake were found on the PC incidence.

## Introduction

### Rationale

PC is the most prevalent cancer in male and the third leading cause of cancer-related death worldwide (1). It has been estimated that 26,730 American men died of PC in 2017 (2). Except for the three already well-established non-modifiable risk factors, age, race, and family history, the etiology of PC remains largely unknown (3, 4). Therefore, it is important to identify effective methods of preventing PC, which may subsequently reduce the substantial burden placed on society by this significant health issue.

Experimental studies suggested that chronic inflammation is involved in the carcinogenesis of PC, especially high-grade PC (5–8). It was demonstrated that tumor cell proliferation and resistance to apoptosis were enhanced through the synthesis of pro-tumor and immunosuppressive cytokines that are present in a chronically inflamed environment. Given the anti-inflammatory and antithrombotic properties of non-steroidal anti-inflammatory drugs (NSAIDs), it is very important to discern their potential role in the development of PC. NSAIDs suppress inflammation and the synthesis of prostaglandin by inhibiting the cyclooxygenase enzyme (COX). Mechanistically, studies verified that NSAIDs, like aspirin, exhibit their chemopreventive effects through both isoforms of the COX enzyme pathway (COX-1 and COX-2). The micrometastasis of PC cells was impaired by the antithrombotic effect of COX-1 inhibition in platelets, which could release pro-angiogenic factors to facilitate the escape of cancer cells from immune surveillance (9, 10). Meanwhile, COX-2 is significantly over-expressed in human PC tumor tissues (2, 11), and the blockage of COX-2 could prevent the production of downstream prostanoids, which contribute to tumorigenesis by promoting cell proliferation, induction, angiogenesis, invasion, and metastasis (12).

Epidemiological studies reported an inverse association between the intake of NSAIDs and the risk of colorectal cancer, gastric cancer, and breast cancer (13–15). Nevertheless, studies on the use of NSAIDs and the risk of PC produced conflicting results (16–19). Although many observational studies revealed a modest inverse association between the use of NSAIDs and PC occurrence, at the same time, other investigations, including several recent meta-analyses, conversely reported no association or even a positive association (18, 20–23). A meta-analysis (from articles up to October 2013) revealed a positive relationship between any type of the use of NSAIDs use and the incidence of PC (20), while another meta-analysis (without language restrictions) demonstrated that NSAIDs did not have either adverse or beneficial effects on the risk of developing PC (22). These two studies were both published around the same time. Interestingly, results regarding the association of the use of NSAIDs with the prevalence of localized PC, advanced PC, and overall PC were also inconsistent. Currently, several large-scale studies performed after the meta-analysis mentioned above was conducted may provide more reliable statistical evidence to help us better understand this issue.

Given the widespread use of NSAIDs, more information is needed to carefully weigh their role in the incidence of PC. Therefore, we performed this meta-analysis to clarify the potential association between the use of NSAIDs and the risk of PC to investigate the sources of variability between studies, which may highlight the importance of considering methods of preventing PC.

### Objectives

This meta-analysis aimed to explore the association between the use of NSAIDs and the risk of total PC, advanced PC and PC with Gleason score ≥7.

### Research question

Does the intake of NSAIDs reduce the risk of total PC, advanced PC and PC with Gleason score ≥7?

## Methods

### Study design

This study was conducted in accordance with the 2015 Preferred Reporting Items for Systematic Review and Meta-Analysis Protocols (PRISMA-P) and the Meta-Analysis of Observational Studies in Epidemiology (MOOSE) guidelines (24, 25).

### Participants, interventions, comparators

We included retrieved articles whose design was a case-control, cohort or cross-sectional study evaluating the association between the use of NSAIDs and the incidence of PC. No restrictions were imposed regarding language.

We included the participants who were exposed to any single NSAID or a mixture of NSAIDs.

We included studies whose results included odds ratios (ORs), relative risks (RRs), hazard ratios (HRs), standardized incidence ratios (SIRs), or incidence rate ratios (IRRs) and 95% confidence intervals (95% CIs), or provided the available raw data needed to calculate the RRs, wherever possible.

### Systematic review protocol

This meta-analysis is registered with the International Prospective Register of Systematic Reviews (PROSPERO registration number: CRD42018090475).

### Search strategy

Ten computerized literature databases were searched systematically by a professional librarian for relevant studies up to December 31, 2017. Medical subject headings (MeSH) in combination with free text searches were used. The full search strategy is presented in Table 1. In particular, those negative studies published in “gray literature,” such as theses, book chapters, and meeting abstracts, were also searched manually. The bibliographies of retrieved articles and previous meta-analyses were also screened to identify additional citations. No exclusion criteria were imposed.

**Table 1:** Search strategy.

**DATABASES USED IN THE SEARCH**	
Medline, EMBASE, Web of science, Cochrane Library, BIOSIS, Scopus, CBM (Chinese Biomedical Literature Database), CNKI (China National Knowledge Infrastructure), WANFANG (Wanfang Database), and CQVIP (Chongqing VIP Database)	
**SEARCH ALGORITHM**	
Search terms #1	“Prostatic Neoplasms”[Mesh] (OR) prostate neoplasm* (OR) prostatic neoplasm* (OR) prostate cancer* (OR) prostatic cancer* (OR) prostate carcinoma* (OR) prostatic carcinoma* (OR) prostate adenocarcinoma* (OR) prostatic adenocarcinoma*
Search terms #2	“Anti-Inflammatory Agents, Non-Steroidal”[Mesh] (OR) NSAID* (OR) non-steroidal anti-inflammatory drugs (OR) non-steroidal anti-inflammatory drugs (OR) non-steroidal anti-inflammatory drugs (OR) non-steroidal anti-inflammatory drugs (OR) non-steroidal anti-inflammatory drugs (OR) non-steroidal anti-inflammatory drugs (OR) non-steroidal anti-inflammatory drugs (OR) non-steroidal anti-inflammatory drugs (OR) non-steroidal anti-inflammatory drugs (OR) non-steroidal anti-inflammatory agents (OR) non-steroidal anti-inflammatory agents (OR) non-steroidal anti-inflammatory agents (OR) non-steroidal anti-inflammatory agents (OR) non-steroidal anti-inflammatory agents (OR) non-steroidal anti-inflammatory agents (OR) non-steroidal anti-inflammatory agents (OR) non-steroidal anti-inflammatory agents (OR) non-steroidal anti-inflammatory agents (OR) “Cyclooxygenase 2 Inhibitors”[Mesh] (OR) Cyclooxygenase-2 inhibitors (OR) Cyclooxygenase 2 inhibitors (OR) Cyclooxygenase2 inhibitors (OR) COX-2 inhibitors (OR) COX 2 inhibitors (OR) COX2 inhibitors (OR) “Aspirin”[Mesh] (OR) aspirin (OR) acetylsalicylic acid (OR) “Celecoxib”[Mesh] (OR) celecoxib (OR) “Diclofenac”[Mesh] (OR) diclofenac (OR) “Diflunisal”[Mesh] (OR) diflunisal (OR) “Etodolac”[Mesh] (OR) etodolac (OR) “Fenoprofen”[Mesh] (OR) fenoprofen (OR) “Flurbiprofen”[Mesh] (OR) flurbiprofen (OR) “Ibuprofen”[Mesh] (OR) ibuprofen (OR) “Indomethacin”[Mesh] (OR) indomethacin (OR) “Ketoprofen”[Mesh] (OR) ketoprofen (OR) “Mefenamic Acid”[Mesh] (OR) mefenamic acid (OR) meloxicam (OR) nabumetone (OR) “Naproxen”[Mesh] (OR) naproxen (OR) “Phenylbutazone”[Mesh] (OR) phenylbutazone (OR) “Piroxicam”[Mesh] (OR) piroxicam (OR) rofecoxib (OR) “Sulindac”[Mesh] (OR) sulindac (OR) tiaprofenic acid (OR) “Tolmetin”[Mesh] (OR) tolmetin (OR) zomepirac (OR) “Acetaminophen”[Mesh] (OR) acetaminophen (OR) paracetamol
Search terms #3	Search terms #1 AND search terms #2

### Data sources, studies sections, and data extraction

Reviews, case reports, letters, commentaries, and animal experimental studies were all excluded. If overlapping study populations were identified, the study with the larger population or greater amount of information was selected for inclusion, but relevant articles with required information were also included. The identification of relevant studies was performed independently by two different authors (JW and XW), and disagreements were resolved through consultation with a third reviewer (HY).

The methodological quality of the included articles was assessed by two independent authors (QW and XW) according to the Newcastle–Ottawa scale (NOS) (for case-control and cohort study) and a modified version of the NOS (for cross-sectional study, Supplementary File S1 in Supplementary Material). When study comparability was evaluated by the NOS, one of the three well-established risk factors (age, race, and family history) was selected as the most important adjusted covariate. Similarly, any of the comorbidities or drugs used simultaneously was chosen as the second most important adjusted factors. “High-quality studies” were defined as having a total NOS score of ≥7, while the others were considered “poor-quality studies.” For each article included, the following information was extracted: the first author's name, year of publication, country, study design, type of controls, numbers of cases and controls (exposure and non-exposure for cohort studies), study period, information source, types of NSAIDs used, definition of NSAIDs uses, adjusted factors, effects estimates as reported or associated raw data and corresponding 95% CIs. Estimates of the association between the intake of NSAIDs and the risk of advanced PC were also extracted. Data were obtained and reviewed independently by two reviewers (ZS and HY), and discrepancies were resolved by group consensus.

### Data analysis

The effect estimates, such as ORs, HRs, SIRs and IRRs, were extracted. Since the absolute risk of PC is low, the above-mentioned measures of association are mathematically approximately equal to the estimates of RRs. Consequently, the pooled RR and its 95% CIs were used to assess the association between the intake of NSAIDs and the risk of PC, making it possible to conduct a comprehensive analysis and to maximize the statistical power (26). If data from different durations of NSAIDs use or different NSAIDs intake levels were available, we selected the data from the longest duration or highest level of intake. Considering that ≥4 or ≥5 years were the most common definition of long-term drugs intake period in the original studies, both were adopted to investigate the pooled effect estimates of long-term drugs intake. Besides, advanced PC was defined as prostatic specific antigen (PSA) ≥20 ng/mL, tumor stage ≥T_2c_N_0_M_0_, or Gleason score ≥7.

Statistical analyses were performed using STATA Statistical Software version 11.0 (STATA Corp, College Station, Texas, USA). The Cochrane *Q*-test and the Higgins *I*^2^-test were used to explore the extent of heterogeneity across the included articles (27, 28). When the *I*^2^-value exceeded 50%, a random-effects model was employed; otherwise, a fixed-effect model was adopted. A χ^2^-based Q test was also performed to check between-study heterogeneity, with *P* < 0.1 indicating statistical significance. Potential sources of heterogeneity were investigated in subgroup analyses, which were based on study design, study quality (total NOS score ≥7), participants, geographic location, dose or duration of drug intake, the sources of drugs, adjusted confounders (numbers of the three main factors and whether they were adjusted for comorbidity or the simultaneous use of other medications), types of effect measures (ORs, RRs, or HRs), information source, and study period. Given that PSA-based screening for PC may be more popular after 2000 than that before 2000. Thus, studies were stratified by study period after 2000 or that before 2000. Publication bias was evaluated by Egger's and Begg's tests. A sensitivity analysis was subsequently conducted to explore whether the pooled result was influenced by individual studies (29, 30).

## Results

### Flow diagram

**Figure 1:**
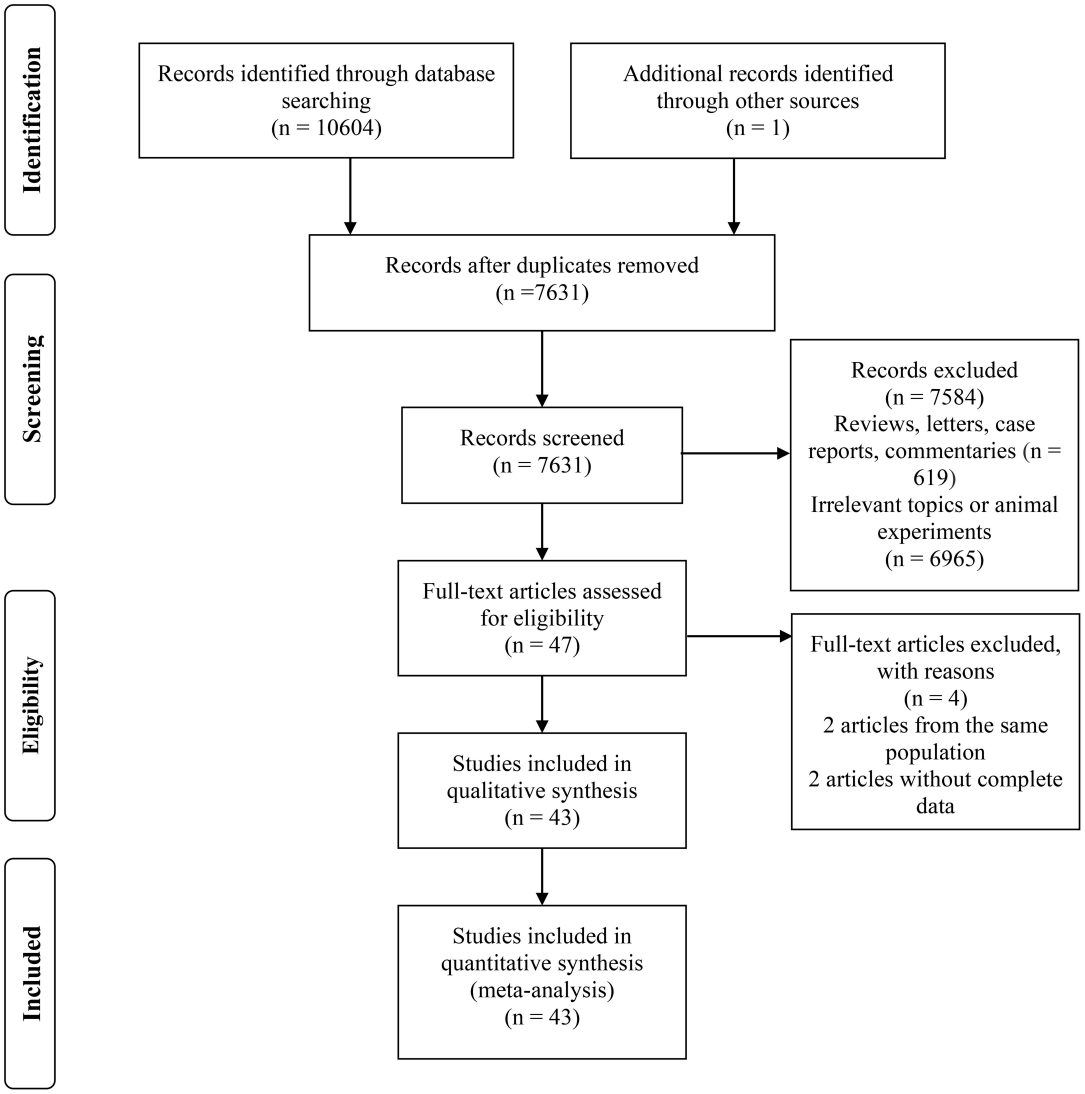
The PRISMA trial flow diagram for identifying and selecting articles.

Figure 1 illustrates the PRISMA trial flow diagram for identifying and selecting articles.

### Study selection and characteristics

A total of 10,604 articles were identified according to the keywords. One article was identified through references and included. After screening titles or abstracts, we identified 47 articles for full-text review. Two articles were excluded due to the lack of complete data needed to evaluate the estimates of the effect of the intake of NSAIDs on PC incidence, and 2 articles were excluded because they had less data than that of another 2 articles from the same populations. Finally, a total of 43 articles were included.

Among the identified articles, there were 19 case-control studies (17, 21, 31–47), 22 cohort studies (18, 19, 23, 48–66), and 2 cross-sectional studies (67, 68). Specifically, most studies (79.07%) were population-based, and more than half of the studies (51.16%) were performed in the USA (23, 31, 35–38, 43, 46, 51–54, 56–58, 61–67). Thirty-two (74.42%) studies also attempted to explore the effect of aspirin intake on the incidence of total PC (17–19, 21, 31–35, 37–42, 45–47, 49, 50, 52–57, 60, 62–65, 67). For advanced PC, 19 studies were included for analysis, which were composed of 8 case-control studies and 11 cohort studies (19, 21, 23, 31–33, 35, 38, 45, 48, 51–54, 58, 62, 65, 66, 68). Moreover, the association between the use of NSAIDs and highly aggressive tumors with Gleason scores ≥7 was investigated in eight studies (19, 21, 32, 48, 51, 53, 54, 68). Information was collected from either databases or questionnaires. Regarding the quality of all the eligible studies, 29 studies (67.44%) were classified as “high-quality studies,” and the others were classified as “poor-quality studies.” Detailed characteristics of the included studies are presented in Table 2 and their NOS score are showed in Supplementary File S2 in Supplementary Material.

**Table 2:** Characteristics of studies included in the meta-analysis.

**References**	**Study design**	**Country**	**Participant**	**Age**	**Drugs**	**Definition of drugs use**	**Cases/exposure**	**Controls/non-exposuree**	**Effect estimate**	**95%CI**	**Outcomes**	**Adjusted factors**	**Study period**	**Information sourceurce**	**Quality score**
(48)	Cohort	Finland	Population-based	55–63	NSAIDs		57,531	21,083	RR	0.75 (0.74–0.75)	Total PC, advanced PC	Age	1995–2009	Database	7
(31)	Case-Control	USA	Population-based	40–90	Aspirin	At least 1 tablet daily in the past 5 years	811	1,023	OR	0.86 (0.71–1.06)	Total PC, advanced PC	Age, BMI, diabetes, education, family history of prostate cancer, race, smoking history, and Tylenol and pain relievers not containing Tylenol or aspirin-containing compounds	2005–2015	Questionnaire	7
(32)	Case-Control	France	Population-based	≤ 75	NSAIDs, Aspirin, non-aspirin NSAIDs	At least once a month	819	879	OR	0.77 (0.61–0.98)	Total PC, advanced PC	Age, family history of cancer at first degree, race, educational level, history of prostatitis, waist to hip ratio	2012–2013	Questionnaire	7
(49)	Cohort	Sweden	Population-based	≥18	Aspirin, non-aspirin NSAIDs				SIRs	0.87 (0.85–0.88)	Total PC	Age	2005–2012	Questionnaire	7
(18)	Cohort	Korea	Population-based	≥40	NSAIDs, Aspirin		1,305	142,565	HR	1.35 (1.14–1.58)	Total PC	Age distribution, sex, insurance eligibility status, Charlson Comorbidity Index, participant's income level and whether or not drug usage and drug types	2004–2013	Database	8
(66)	Cohort	USA	Health professionals	40–84	Aspirin	>3 tablets/week for at least 1 year	12,454	9,496	HR	0.68 (0.52–0.89)	Advanced PC	Age, race, BMI, height, smoking, hypertension, and type 2 diabetes	1981/82–2009	Questionnaire	7
(21)	Case-Control	Denmark	Population-based	70	Aspirin, non-aspirin NSAIDs	≥ 2 redeemed prescriptions on separate days, more than 1 year prior to the index date	35,600	177,992	OR	0.94 (0.91–0.97)	Total PC, advanced PC	Age and residence, use of high-dose aspirin, 5-alpha reductase inhibitors, statins, selected cardiovascular drugs, and antidepressants or neuroleptics, history of diabetes mellitus; educational level; income; and mutual adjustment for use of low-dose aspirin or non-aspirin NSAIDs	2000–2012	Database	7
(67)	Cross-sectional	USA	Population-based	≥20	Aspirin	At least 3 times a week	2,457,316	104,026,095	OR	0.60 (0.38–0.94)	Total PC	Age, race/ethnicity, education, US citizen, and cancer-related health beliefs, insurance, family income, region of residence, regular finasteride use, regular use of non-aspirin NSAIDs or COX-2 inhibitors, and antidiabetic drug use, family history of prostate cancer, smoking status, alcohol drinking status, frequency of vigorous physical activity, nutritional status, health status, numbers of PSA tests performed during the past 5 years, BMI, and selfreported diabetes mellitus	1987–2010	Questionnaire	7
(50)	Cohort	Italy	Population-based	≥18	Aspirin		7,747	5,706	HR	0.64 (0.48–0.86)	Total PC	Age, presence of obesity, smoking, alcohol abuse or related diseases, Charlson Comorbidity Index, benign prostatic hypertrophy, numbers of PSA requests, use of ACE inhibitors, NSAIDs, statins, alpha-adrenoreceptor antagonists, testosterone 5-alpha reductase inhibitors, immunosuppressive drugs	2002–2013	Database	7
(23)	Cohort	USA	Population-based	40–75	Aspirin		3,748	3,823	RR	0.97 (0.85–1.1)	Advanced PC	Race, height, BMI, family history of cancer, physical examination in the past 2 years, history of colonoscopy or sigmoidoscopy, smoking, physical activity, alcohol intake, current multivitamin use, total energy intake, red and processed meat intake, folate intake, calcium intake, and Alternate Healthy Eating Index 2010 prostate-specific antigen test in the past 2 years	1986–2012	Questionnaire	8
(51)	Cohort	USA	Population-based	50–75	NSAIDs		3,221	3,169	OR	0.92 (0.78–1.08)	Total PC, advanced PC	Age, race, baseline PSA, prostate volume, DRE findings, BMI, treatment arm, geographic region, smoking, cardiovascular disease, diabetes, alcohol use, statin medication, and hypertension hypertension	2003–2007	Database	6
(19)	Cohort	Sweden	Population-based	71.6	Aspirin		26,409	177,822	OR	1.115 (0.995–1.25)	Total PC, advanced PC	Age, natural log-transformed PSA concentration, PSA quotient, Charlson Comorbidity Index, educational level, use of aspirin, use of statin and use of antidiabetic medication	2003–2012	Database	7
(33)	Case-Control	Finland	Population-based	20–96	Aspirin, NSAIDs		24,657	24,657	OR	0.95 (0.88–1.0)	Total PC, advanced PC	Age and simultaneous use of other medications (cholesterol lowering drugs, anti-diabetic drugs, antihypertensive drugs and benign prostatic hyperplasia medication)	1995–2002	Database	8
(52)	Cohort	USA	Population-based	62.8	Aspirin, non-aspirin NSAIDs		15,893	13,539	RR	0.92 (0.85–0.99)	Total PC, advanced PC	Race, study center, family history of prostate cancer, the number of screening exams, aspirin use (for non-aspirin NSAIDs only), and non-aspirin NSAIDs use (for aspirin only)	1993–2009	Questionnaire	7
(53)	Cohort	USA	Health professionals	40–75	Aspirin	≥ 2 days/week	18,570	24,494	HR	0.94 (0.87–1.02)	Total PC, advanced PC	Age, period, family history, ethnic, height, BMI, tomato sauce, vigorous physical activity, smoking, vitamin D, fish, red meat, cholesterol-lowering drugs and total kcal	1988–2006	Questionnaire	7
(17)	Case-Control	UK	Population-based	50–69	NSAIDs, Aspirin, non-aspirin NSAIDs		1,016	5,043	OR	1.24 (1.06–1.46)	Total PC	Age, family history of prostate cancer, BMI and self-reported diabetes status	2001–2008	Questionnaire	7
(34)	Case-Control	Canada	Population-based	≥40	NSAIDs, Aspirin, non-aspirin NSAIDs		9,007	35,891	OR	0.87 (0.80–0.94)	Total PC	Ever visited a urologist 1–11 years prior, volume of family physician visits in the 5 years prior to the index date and, when appropriate, for use of other NSAIDs classes, Binary variable with 1 indicating whether at any point prior to the index date a subject had a physician visit for BPH, prostatitis, other disorders of prostate or any point during the 11 years prior to the index date, a subject received at least one prescription for finasteride or an a-blocker or had prostatic ablation or resection, or testing of prostatic secretions	1985–2000	Database	7
(54)	Cohort	USA	Population-based	50–76	Aspirin, non-aspirin NSAIDs		10,767	23,265	HR	0.98 (0.87–1.09)	Total PC, advanced PC	Age, race, education, BMI, multivitamin use, PSA test in the past 2 years, benign prostate biopsy, enlarged prostate, family history of prostate cancer, diabetes, coronary artery disease, osteoarthritis, rheumatoid arthritis, chronic joint pain, chronic headaches, and migraines	2000–2007	Questionnaire	7
(35)	Case-Control	USA	Population-based	35–74	Aspirin, non-aspirin NSAIDs	At least once per week for a period of 3 months or longer	1,000	942	OR	0.82 (0.68–0.99)	Total PC, advanced PC	Age at reference date, race, prostate cancer screening within 5 years before reference date	2002–2005	Questionnaire	7
(36)	Case-Control	USA	Hospital-based	40–79	NSAIDs		1,367	2,007	OR	0.9 (0.4–2.2)	Total PC	Age, study center, interview year and BMI, alcohol use, pack-years of smoking, race, family history of PC, number of doctor visits made 2 years before hospital admission and education	1992–2008	Questionnaire	6
(55)	Cohort	Netherlands	Population-based	≥ 55	NSAIDs, Aspirin, non-aspirin NSAIDs				HR	1.02 (0.76–1.37)	Total PC	Age, BMI, C-reactive protein level and pack years of smoking	1989–1993	Questionnaire	7
(56)	Cohort	USA	Population-based	≥50	Aspirin		53,573	16,237	RR	0.81 (0.7–0.94)	Total PC	Age, race, education, smoking, BMI, physical activity level, history of PSA testing, history of colorectal endoscopy, use of non-aspirin NSAIDs, and history of heart attack, diabetes and hypertension etes, and hypertension	1992–2001	Questionnaire	8
(37)	Case-Control	USA	Population-based		Aspirin	More than 1 pill per week for more than 1 year	229	285	OR	0.52 (0.29–0.93)	Total PC	Age, body mass, family history, smoking, alcohol intake	2002–2004	Database	6
(68)	Cross-sectional	Canada	Population-based	59–71	NSAIDs	Daily	494	805	OR	0.71 (0.48–1.03)	Total PC, advanced PC	age, family history of prostate cancer, history of ischaemic heart disease, intake of acetaminophen, reasons for referral and prostate volume	1999–2003	Questionnaire	7
(38)	Case-Control	USA	Hospital-based	67.1	Aspirin	At least once a week for at least 6 months	1,029	1,029	OR	1.05 (0.89–1.25)	Total PC, advanced PC	Age, education, family history of prostate cancer, cigarette smoking, race, and BMI	1982–1998	Questionnaire	6
(39)	Case-Control	Italy	Hospital-based	46–74	Aspirin	At least once a week for more than 6 months	1,261	1,131	OR	1.10 (0.81–1.50)	Total PC	Age, study center, education and family history of prostate cancer	1991–2002	Questionnaire	6
(47)	Case-Control	Canada	Hospital-based	≥65	NSAIDs, Aspirin	≥ 1 prescription more than 4 months	2,025	2,150	OR	0.71 (0.58–0.86)	Total PC	Age and finasteride use	1999–2002	Questionnaire	6
(58)	Cohort	USA	Population-based	≥ 50	NSAIDs		41,094	29,050	RR	0.95 (0.86–1.05)	Total PC, advanced PC	Age, race, diabetes, history of heart attack, history of PSA testing, education, and family history of prostate cancer in a brother or father	1992–2001	Questionnaire	8
(57)	Cohort	USA	Population-based	70	NSAIDs, Aspirin, non-aspirin NSAIDs				RR	0.71 (0.49–1.02)	Total PC	Age and analgesic drugs	1980–2004	Questionnaire	8
(40)	Case-Control	UK	Hospital-based	50–79	Aspirin, non-aspirin NSAIDs	Current use	2,183	10,000	OR	0.70 (0.61–0.79)	Total PC	Age, calendar year, prior BPH history, number of visits to general practitioners, referrals, hospitalizations	1995–2001	Database	6
(41)	Case-Control	Canada	Population-based	73–79	NSAIDs, Aspirin	At least 325 mg daily	2,221	11,105	OR	1.14 (0.85–1.54)	Total PC	Age and recent medical contacts	1993–1995	Database	6
(60)	Cohort	Denmark	Population-based	70	Aspirin		15,058		SIR	1.1 (1.0–1.3)	Total PC	Age	1989–1995	Questionnaire	7
(59)	Cohort	Denmark	Population-based	47.2	Non-aspirin NSAIDs		78,562		SIR	1.3 (1.2–1.5)	Total PC		1989–1995	Questionnaire	6
(65)	Cohort	USA	Health professionals	40–75	Aspirin				RR	1.10 (1.01–1.19)	Total PC, advanced PC	Age, time period, BMI at age 21, height, pack-years of smoking in the previous decade, family history of prostate cancer, vigorous physical activity, intake of total energy, calcium, fructose, tomato-based foods, red meat, fish, supplemental vitamin E, linoleic acid, and α-linolenic acid	1986–1996	Questionnaire	6
(62)	Cohort	USA	Population-based	18–84	Aspirin	More than 6 aspirin almost every day	2,466	87,634	RR	0.76 (0.60–0.98)	Total PC, advanced PC	Birth year, education, race, and number of health checkups	1964–1973	Questionnaire	7
(61)	Cohort	USA	Community-based	56.7–70.9	NSAIDs	Daily	592	861	OR	0.45 (0.28–0.73)	Total PC		1990–1996	Questionnaire	5
(42)	Case-Control	France	Population-based	66.8	NSAIDs, Aspirin, non-aspirin NSAIDs	Any of these medications some time during the 5 years before the interview	639	659	RR	0.90 (0.86–0.93)	Total PC	Age, farming, ethnic origin, frequency of red meat and red wine consumption, aspirin and non-aspirin NSAIDs, finasteride and urological center	1999–2000	Questionnaire	8
(44)	Case-Control	UK	Population-based		NSAIDs	Prescribed NSAIDs in 13–36 months before diagnosis of case	1,813	5,354	OR	1.33 (1.07–1.64)	Total PC	Age and smoking	1990–1993	Database	7
(43)	Case-Control	USA	Population-based	64	NSAIDs		417	420	OR	0.34 (0.2–0.58)	Total PC	Age, race, and other factors	1992–1995	Questionnaire	6
(45)	Case-Control	New Zealand	Population-based	40–80	NSAIDs, Aspirin, non-aspirin NSAIDs		317	480	OR	0.88 (0.64–1.20)	Total PC, advanced PC	Age, socio-economic status, total polyunsaturated fat consumption, α-linolenic acid and ratio of dietary n-6: long-chain n-3 polyunsaturated fatty acids	1996–1997	Questionnaire	6
(46)	Case-Control	USA	Hospital-based	69.6	Aspirin		319	189	OR	1.6 (0.82–3.11)	Total PC	Age, race, and history of coronary heart disease, diabetes	1984–1986	Questionnaire	7
(63)	Cohort	USA	Population-based	25–74	Aspirin				IRR	0.95 (0.66–1.35)	Total PC	Age, race, education, socioeconomic status, BMI, alcohol consumption, and arthritis	1971–1987	Questionnaire	7
(64)	Cohort	USA	Population-based	73	Aspirin		1,561	3,490	RR	0.95 (0.84–0.97)	Total PC	Age	1981–1988	Questionnaire	6

### Synthesized findings

#### Intake of any NSAIDs and total or advanced PC risk

**Figure 2:**
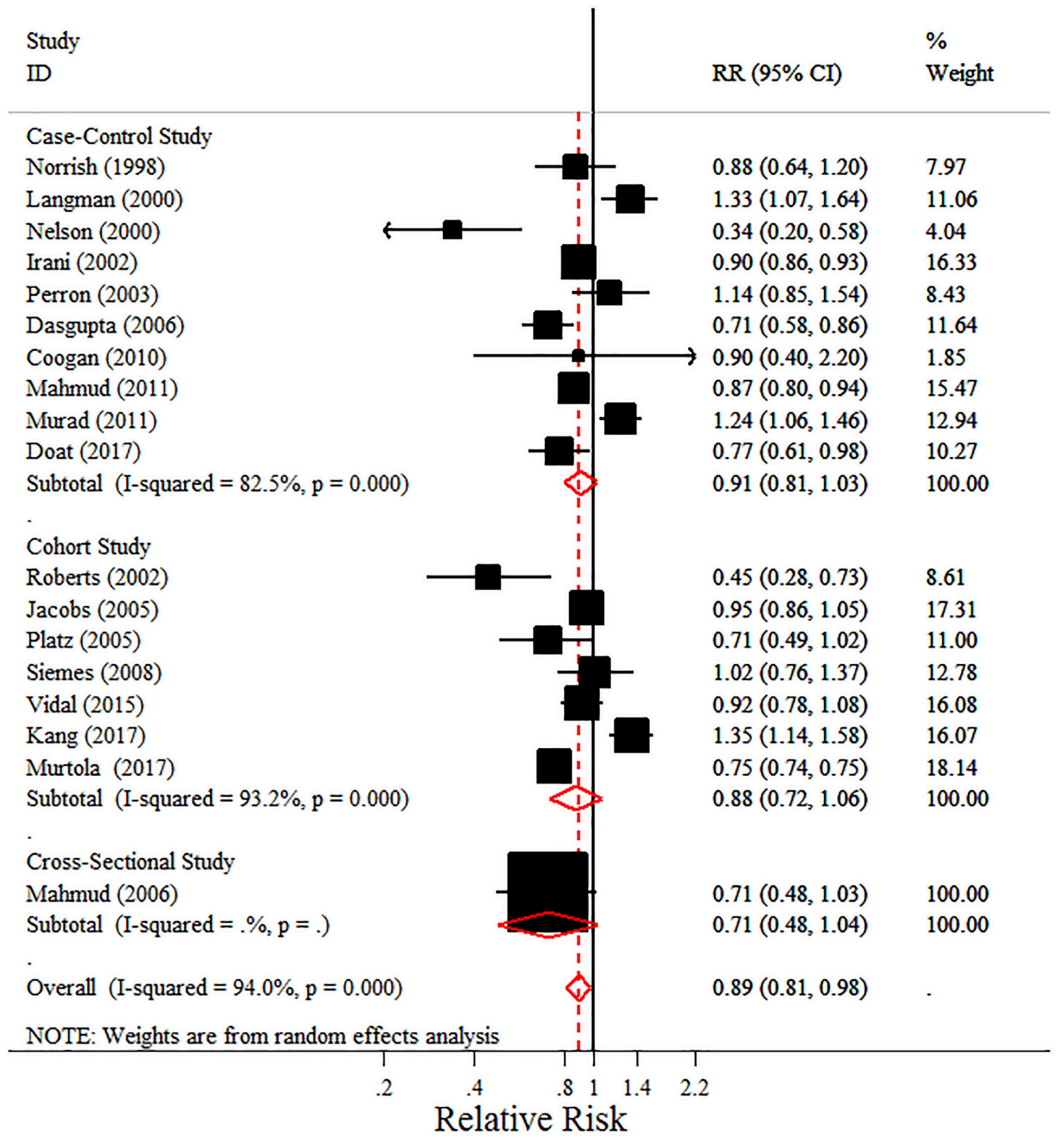
Forest plot and meta-analysis of the association between the intake of any NSAIDs and the risk of prostate cancer.

Unlike previously conducted meta-analyses, this meta-analysis found a protective effect of the intake of any NSAIDs on PC risk due to the inclusion of recent research (pooled RR = 0.89, 95% CI = 0.81–0.98) (Figure 2). However, there was some evidence of heterogeneity (*I*^2^ = 94.00%, *P* < 0.001). Possible reasons for that heterogeneity were explored in the subgroup analyses. In addition, a non-significant decreased risk was detected for advanced PC or PC with Gleason score ≥7, though the pooled RRs were <1.

In the subgroup analyses, we observed that the intake of NSAIDs was associated with a decreased PC risk in hospital-based studies and studies from North America (pooled RR 0.719, 95% CI = 0.593–0.871, *I*^2^ = 0.00%; pooled RR 0.797, 95% CI = 0.698–0.910, *I*^2^ = 72.10%, respectively). Interestingly, long-term intake of NSAIDs (≥5 years rather than ≥4 years) was associated with an 11.8% reduction in PC incidence, with little evidence of heterogeneity (pooled RR = 0.882, 95% CI = 0.785–0.991, *P* = 0.035; *I*^2^ = 27.40%, *P* = 0.248). Notably, no significantly beneficial effects were found in the “High-quality studies” on total PC. However, when we restricted our analysis to studies adjusting for comorbidities, the association did not remain and there was no significance when the studies were stratified by the numbers of the three main factors, though the pooled RR was also <1 (Table 3).

**Table 3:** Stratified pooled effects and 95% confidence intervals of NSAIDs intake and prostate cancer risk.

**Study characteristics**		**Number of studies**	**Effect estimates (95% CI)**	***P* value**	**Effect model**	**Heterogeneity**	
						***I*^2^ (%)**	***P* value**
**STUDIES OF TOTAL PROSTATE CANCER**							
**NSAIDs**							
Study design	Case–control studies	10	0.913 (0.807, 1.032)	0.147	Random	82.50	<0.001
	Cohort studies	7	0.877 (0.722, 1.065)	0.185	Random	93.20	<0.001
Study quality	High quality studies	11	0.950 (0.847, 1.066)	0.381	random	96.10	<0.001
	Poor quality studies	7	0.742 (0.580, 0.950)	0.018	random	76.40	<0.001
Participant	Population-based studies	15	0.927 (0.836, 1.028)	0.15	Random	95.00	<0.001
	Hospital-based studies	2	0.719 (0.593, 0.871)	0.001	Fixed	0.00	0.595
Country	Studies from North America	10	0.797 (0.698, 0.910)	<0.001	Random	72.10	<0.001
	Studies from Europe	6	0.960 (0.829, 1.111)	0.582	Random	97.00	<0.001
	Studies from other countries	2	1.114 (0.734, 1.691)	0.612	Random	82.20	0.018
Duration	Long-time NSAIDs use (≥4 years)	6	1.023 (0.833, 1.255)	0.83	Random	72.40	0.003
	Long-time NSAIDs use (≥5 years)	4	0.882 (0.785, 0.991)	0.035	Fixed	27.40	0.248
Effect estimates	Effect estimate OR	13	0.878 (0.786, 0.980)	0.021	Random	80.30	<0.001
	Effect estimate RR	2	0.868 (0.667, 1.130)	0.294	Random	55.70	0.133
	Effect estimate HR	2	1.207 (0.922, 1.579)	0.171	Random	62.40	0.103
NSAIDs source	Prescription database	7	0.865 (0.663, 1.129)	0.286	Random	93.70	0.109
Adjusted factors	Less than 2 of three main adjusted factors	10	0.905 (0.775, 1.056)	0.205	Random	91.90	<0.001
	Equal or more than 2 of three main adjusted factors	8	0.887 (0.782, 1.006)	0.062	Random	78.30	<0.001
Comorbidity	Did not adjust for comorbidity	13	0.892 (0.799, 0.995)	0.04	Random	94.30	<0.001
	Adjusted for comorbidity	4	0.976 (0.824, 1.157)	0.781	Random	74.90	0.008
Concomitant use of medication	Did not adjust for concomitant use of other medications	9	0.851 (0.700, 1.034)	0.105	Random	92.50	<0.001
	Adjusted for concomitant use of other medications	9	0.911 (0.820, 1.013)	0.085	Random	77.50	<0.001
Information source	Questionnaires	12	0.826 (0.732, 0.933)	0.002	Random	77.30	<0.001
	Database	6	1.016 (0.838, 1.233)	0.871	Random	95.30	<0.001
Study period	Study period before 2000	8	0.895 (0.780, 1.027)	0.114	Random	81.40	<0.001
	Study period after 2000	4	1.054 (0.831, 1.335)	0.666	Random	86.10	<0.001
**Aspirin**							
Study design	Case–control studies	16	0.914 (0.868, 0.961)	0.001	Random	71.70	<0.001
	Cohort studies	17	0.940 (0.887, 0.996)	0.037	Random	81.70	<0.001
Study quality	High quality studies	24	0.942 (0.906, 0.979)	0.002	Random	78.40	<0.001
	Poor quality studies	10	0.870 (0.771, 0.981)	0.024	Random	83.40	<0.001
Participant	Population-based studies	28	0.934 (0.899, 0.971)	0.001	Random	80.20	<0.001
	Hospital-based studies	5	0.927 (0.756, 1.137)	0.469	Random	80.70	<0.001
Country	Studies from North America	20	0.906 (0.857, 0.958)	0.001	Random	66.50	<0.001
	Studies from Europe	12	0.938 (0.888, 0.991)	0.024	Random	87.40	<0.001
	Studies from other countries	2	1.017 (0.935, 1.107)	0.691	Fixed	15.80	0.276
Dose	Daily aspirin use (≥ 1/day)	7	0.875 (0.792, 0.967)	0.009	Random	64.30	0.01
Duration	Long-time aspirin use (≥ 4 years)	15	0.823 (0.571, 1.186)	0.295	Random	99.10	<0.001
	Long-time aspirin use (≥ 5 years)	11	0.792 (0.514, 1.219)	0.288	Random	99.20	<0.001
Effect estimates	Effect estimate OR	19	0.916 (0.870, 0.963)	0.001	Random	73.60	<0.001
	Effect estimate RR	7	0.921 (0.843, 1.007)	0.069	Random	73.00	0.001
	Effect estimate HR	5	0.950 (0.862, 1.047)	0.301	Random	61.50	0.034
Aspirin source	Prescription database	13	0.936 (0.878, 0.996)	0.0038	Random	90.20	0.009
Adjusted factors	Less than 2 of three main adjusted factors	15	0.919 (0.870, 0.971)	0.003	Random	86.20	<0.001
	Equal or more than 2 of three main adjusted factors	19	0.934 (0.888, 0.983)	0.009	Random	59.30	0.001
comorbidity	Did not adjust for comorbidity	23	0.933 (0.892, 0.976)	0.003	Random	83.60	<0.001
	Adjusted for comorbidity	11	0.903 (0.835, 0.976)	0.01	Random	59.40	0.006
Concomitant use of medication	Did not adjust for concomitant use of other medications	18	0.941 (0.876, 1.011)	0.095	Random	79.70	<0.001
	Adjusted for concomitant use of other medications	16	0.925 (0.888, 0.963)	<0.001	Random	69.20	<0.001
Information source	Questionnaires	24	0.937 (0.898, 0.978)	0.003	Random	74.50	<0.001
	Database	10	0.892 (0.824, 0.965)	0.005	Random	84.80	<0.001
Study period	Study period before 2000	12	0.978 (0.920, 1.040)	0.479	Random	67.80	<0.001
	Study period after 2000	12	0.926 (0.871, 0.986)	0.016	Random	82.50	<0.001
**NA-NSAID**							
Study design	Case–control studies	8	1.002 (0.881, 1.140)	0.978	Random	88.60	<0.001
	Cohort studies	7	1.001 (0.866, 1.157)	0.992	Random	89.10	<0.001
Study quality	High quality studies	12	0.966 (0.867, 1.076)	0.524	Random	95.2	<0.001
	Poor quality studies	3	1.219 (1.122, 1.325)	0.001	Fixed	45.30	0.16
Participant	Population-based studies	15	1.001 (0.908, 1.103)	0.987	Random	94.50	<0.001
Country	Studies from North America	6	0.932 (0.886, 0.981)	0.007	Fixed	36.30	0.165
	Studies from Europe	8	1.036 (0.900, 1.192)	0.624	Random	97.00	<0.001
Dose	Daily NA-NSAIDS use (≥ 1/day)	2	0.975 (0.790, 1.203)	0.813	Fixed	0.00	0.773
Duration	Long-time NA-NSAIDS use (≥ 4 years)	6	1.080 (1.079, 1.080)	<0.001	Fixed	0.00	0.451
	Long-time NA-NSAIDS use (≥ 5 years)	3	1.080 (1.079, 1.080)	<0.001	Fixed	30.90	0.235
Effect estimates	Effect estimate OR	8	1.002 (0.881, 1.140)	0.978	Random	88.60	<0.001
	Effect estimate RR	3	0.985 (0.889, 1.092)	0.776	Fixed	0.00	0.487
	Effect estimate HR	2	1.010 (0.897, 1.138)	0.87	Fixed	0.00	1
NA-NSAIDs source	Prescription database	5	1.046 (0.895, 1.223)	0.574	Random	98.30	0.030
Adjusted factors	Less than 2 of three main adjusted factors	9	0.995 (0.873, 1.134)	0.934	Random	96.70	<0.001
	Equal or more than 2 of three main adjusted factors	6	1.015 (0.945, 1.090)	0.68	Fixed	43.90	0.113
Comorbidity	Did not adjust for comorbidity	12	0.981 (0.878, 1.097)	0.741	Random	95.60	<0.001
	Adjusted for comorbidity	3	1.048 (0.948, 1.158)	0.358	Fixed	47.10	0.151
Concomitant use of medication	Did not adjust for concomitant use of other medications	10	1.029 (0.899, 1.178)	0.681	Random	88.20	<0.001
	Adjusted for concomitant use of other medications	5	0.951 (0.812, 1.113)	0.531	Random	92.90	<0.001
Information source	Questionnaires	12	0.987 (0.880, 1.106)	0.819	Random	83.80	<0.001
	Database	3	1.041 (0.869, 1.245)	0.664	Random	95.70	<0.001
Study period	Study period before 2000	5	0.985 (0.782, 1.239)	0.895	Random	88.80	<0.001
	Study period after 2000	6	1.005 (0.857, 1.177)	0.955	Random	97.60	<0.001
**STUDIES OF ADVANCED PROSTATE CANCER**							
Drugs	NSAIDs intake	7	0.906 (0.702, 1.168)	0.445	Random	94.50	<0.001
	Asprin intake	17	0.909 (0.875, 0.945)	<0.001	Fixed	16.20	0.264
	NA-NSAIDs intake	7	1.030 (0.988, 1.074)	0.161	Fixed	0.00	0.803
**STUDIES OF PROSTATE CANCER WITH GLEASON SCORE ≥7**							
Drugs	NSAIDs intake	4	0.868 (0.715, 1.053)	0.152	Random	50.40	0.109
	Aspirin intake	6	0.918 (0.875, 0.964)	0.001	Fixed	28.40	0.222
	NA-NSAIDs intake	3	1.032 (0.988, 1.077)	0.156	Fixed	0.00	0.543

#### Intake of aspirin and total or advanced PC risk

**Figure 3:**
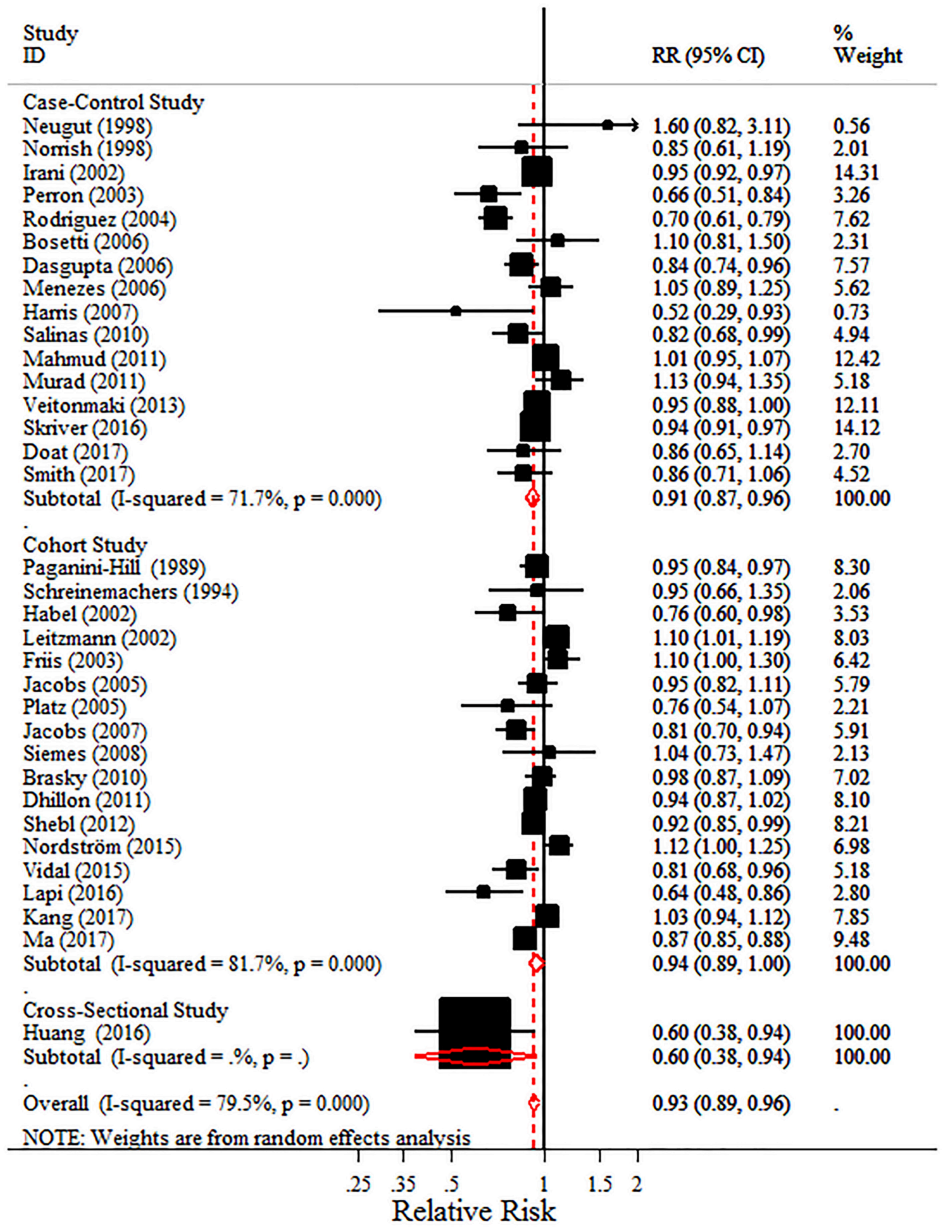
Forest plot and meta-analysis of the association between the intake of aspirin and the risk of prostate cancer.

Considering the pharmacology of aspirin differ from that of other NSAIDs, the meta-analyses for aspirin and non-aspirin NSAIDs (NA-NSAIDs) were also conducted, respectively. The 34 studies that evaluated aspirin intake and total PC risk showed a 7.0% risk reduction of PC with aspirin intake (pooled RR = 0.93, 95% CI = 0.89–0.96) and displayed considerable heterogeneity (*I*^2^ = 79.5%, *P* < 0.001) (Figure 3). Especially, Table 3 shows that with aspirin intake, the risks of advanced PC and PC with Gleason score ≥7 were lower than that of total PC (pooled RR = 0.909, 95% CI = 0.875–0.945; pooled RR = 0.918, 95% CI = 0.875–0.964, respectively) with little heterogeneity (*I*^2^ = 16.20 and 28.40%, respectively).

In the subgroup analyses, a similar negative trend of PC risk with aspirin intake was detected regardless of study design (for case-control studies, pooled RR = 0.914, 95% CI = 0.868 −0.961; for cohort studies, pooled RR = 0.940, 95% CI = 0.887–0.996), quality of studies (for high quality studies, pooled RR = 0.942, 95% CI = 0.906–0.979; for poor quality studies, pooled RR = 0.870, 95% CI = 0.771–0.981), geographic region (for studies from North America, pooled RR = 0.906, 95% CI = 0.857–0.958; for studies from Europe, pooled RR = 0.938, 95% CI = 0.888–0.991), information source (information collected from questionnaires, pooled RR = 0.937, 95% CI = 0.898–0.978; information collected from databases, pooled RR = 0.892, 95% CI = 0.824–0.965), comorbidities, the source of aspirin, and the number of the three main adjusted factors. Contrary to a recent study, it was the daily dose (≥1 pill/day) not the long-term intake of aspirin (≥4 years or ≥5 years) that was associated with reduced PC incidence (pooled RR = 0.875, 95% CI = 0.792–0.967). In addition, we summarized the final pooled effects of aspirin intake from 16 studies which have adjusted for the concomitant use of other medications and found a lower risk of PC (pooled RR = 0.925, 95% CI = 0.888–0.963), compared with the 7.0% risk reduction of PC among all aspirin users. Interestingly, studies performed after the year 2000 showed a negative association unlike those performed before 2000 (pooled RR = 0.926, 95% CI = 0.871–0.986), and the subgroup analysis on ORs also demonstrated a similar negative trend (pooled RR = 0.916, 95% CI = 0.870–0.963) (Table 3).

#### Intake of NA-NSAIDs and total or advanced PC risk

**Figure 4:**
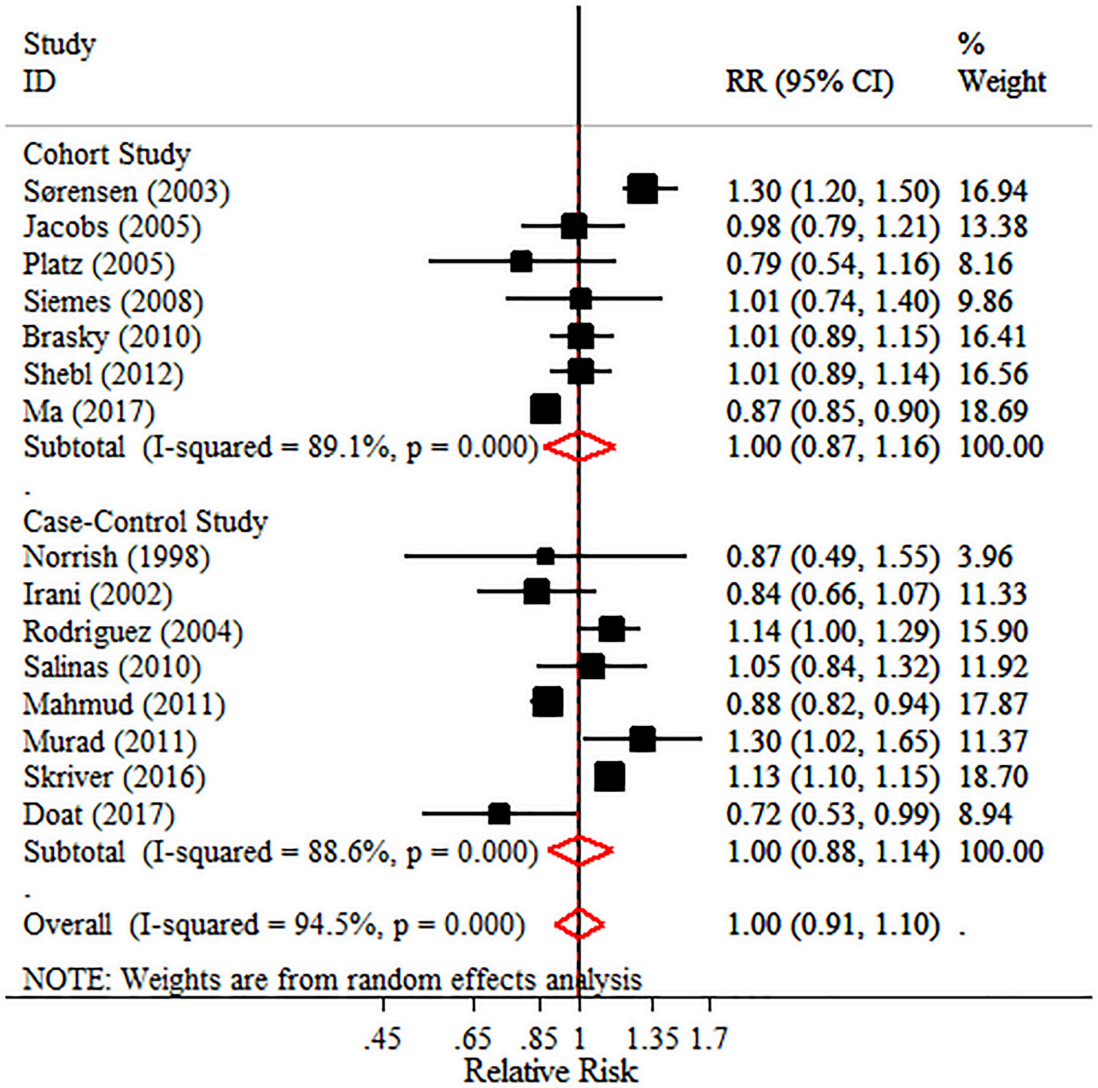
Forest plot and meta-analysis of the association between the intake of NA-NSAIDs and the risk of prostate cancer.

The pooled effects for non-aspirin NSAIDs demonstrated no significantly adverse or beneficial effects on total PC, advanced PC, or PC with Gleason score ≥7. However, all pooled RRs were >1 (Figure 4 and Table 3). Notably, results in the subgroup analyses were not consistent. A decreased PC risk was observed in studies from North America (pooled RR = 0.932, 95% CI = 0.886–0.981), while the long-term intake of non-aspirin NSAIDs (≥4 years or ≥5 years) may be a potential risk factor in PC incidence, with little heterogeneity (*I*^2^ = 0.00 and 30.90%, respectively). Moreover, a non-significant decreased risk was detected in “high quality studies,” though an adverse effect of non-aspirin NSAIDs on total PC was observed in “poor quality studies.” The detailed data are shown in Table 3.

### Risk of bias

**Figure 5:**
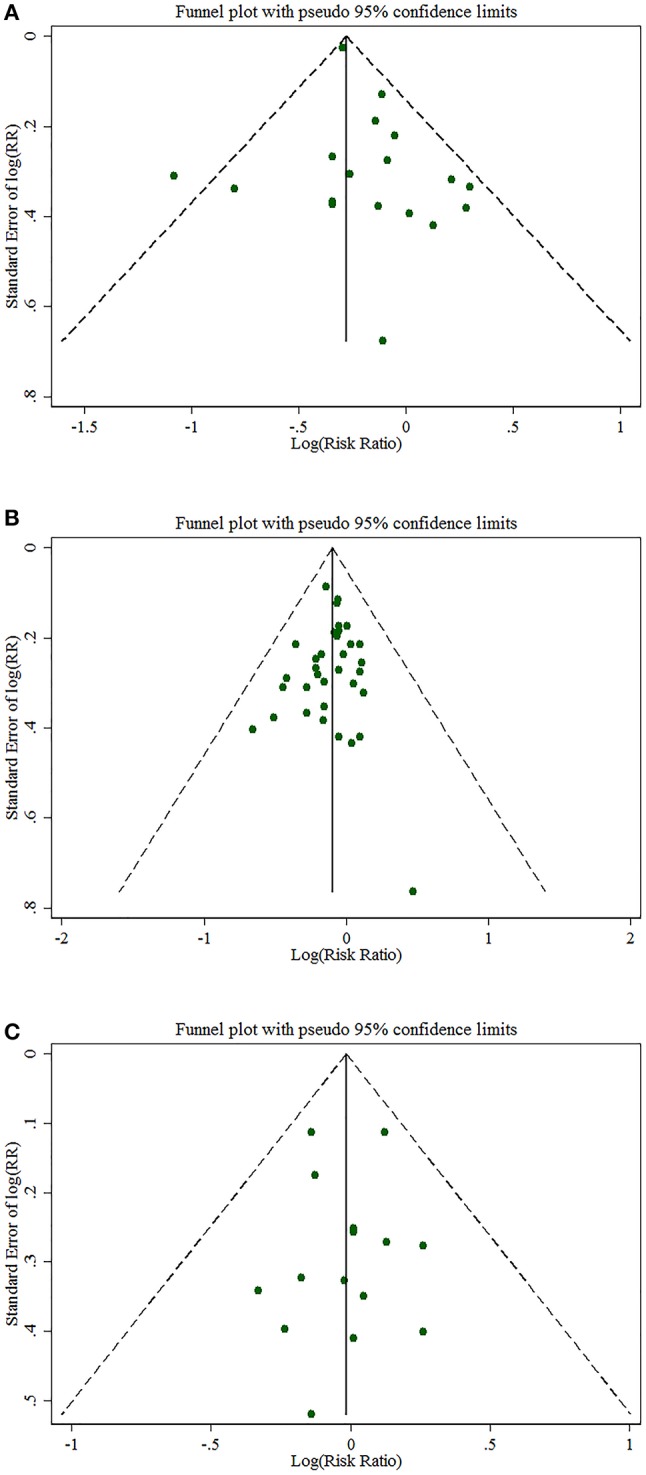
Funnel plots of Begg's test **(A)** for the intake of any NSAIDs, **(B)** for the intake of aspirin, **(C)** for the intake of NA-NSAIDs.

**Figure 6:**
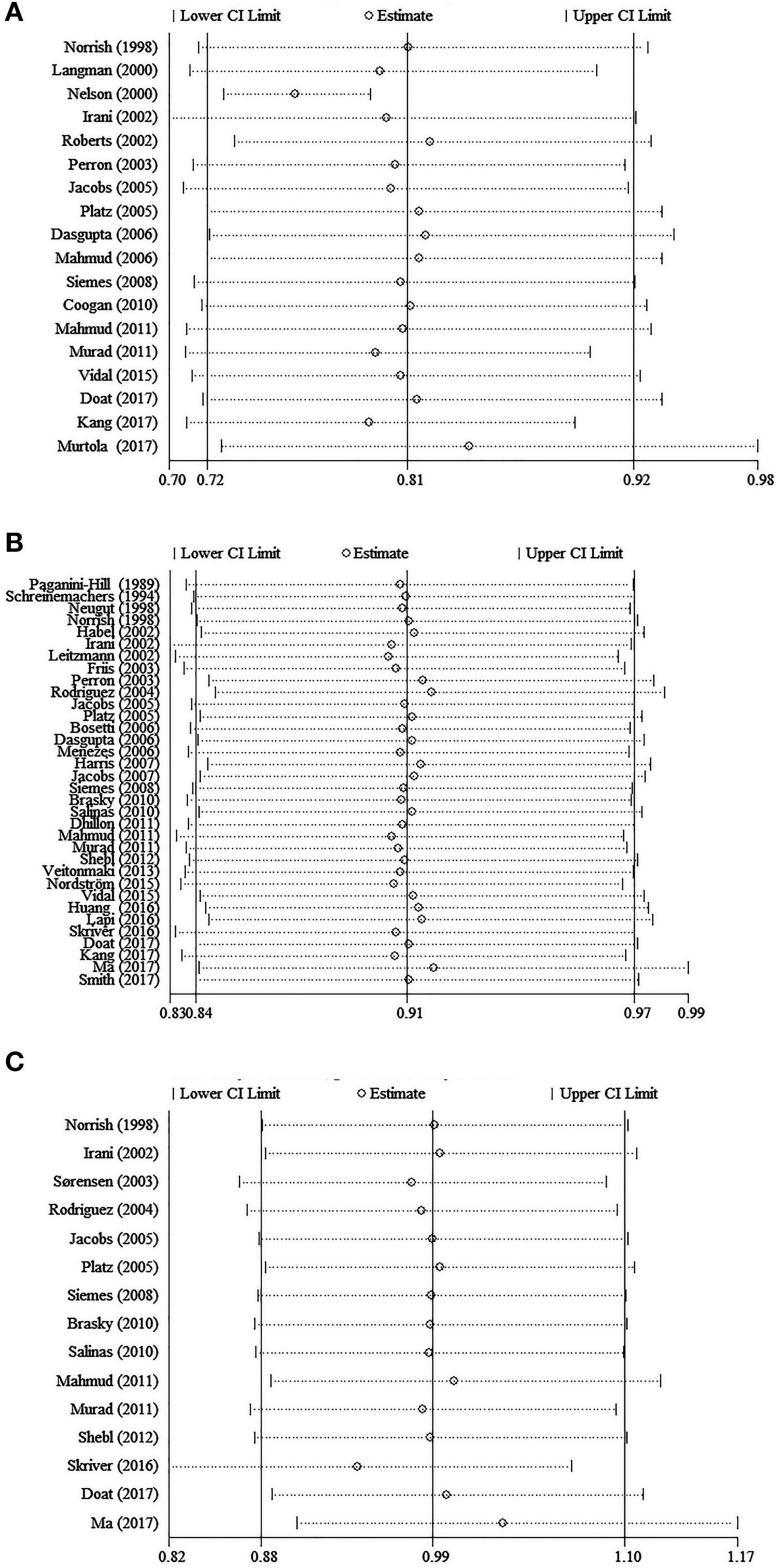
Sensitivity analyses for the pooled risk of prostate cancer **(A)** for the intake of any NSAIDs, **(B)** for the intake of aspirin, **(C)** for the intake of NA-NSAIDs.

No such publication bias was detected by either Begg's or Egger's test (for the intake of any NSAIDs, *p* = 0.185; for the intake of aspirin, *p* = 0.537; for the intake of non-aspirin NSAIDs, *p* = 0.953, respectively) (Figure 5). In sensitivity analyses, none of the individual studies substantially altered the pooled effect estimates for drugs intake on PC incidence (Figure 6). Age, race, and family history of participants were well-established risk factors for PC. In the individual studies, analyses should be systematically adjusted for the three risk factors. In this meta-analysis, most of the included individual studies (93.62%) take at least one of the three risk factors into account. In addition, based on the number of the three main adjusted factors included, subgroup analyses were performed. The NOS and a modified version of the NOS were used to assess the quality of case-control or cohort study and cross-sectional study respectively. More than half of the studies (67.44%) were considered as high-quality studies. Meta-analysis of only these high-quality studies revealed similar risk reduction of PC with aspirin intake, while no significant effects of NSAIDs or non-aspirin NSAIDs intake were found on the PC incidence. Differences in the definitions of drug intake, ages of participants, sample sizes of studies, comorbidities, simultaneous use of other medications and information source of the included individual studies could result in some selection bias, confounding bias and information bias, which may be interpreted by subgroup analyses to some extent.

## Discussion

### Summary of main findings

To the best of our knowledge, this is the first meta-analysis that attempted to explore the association between PC incidence and the dose and duration of the intake of NSAIDs, which could be the most important factors affecting the effect of NSAIDs on PC and which have not been addressed by previous meta-analyses. In our meta-analysis of 43 studies, we observed that the intake of any NSAIDs and the intake of aspirin were inversely related to PC incidence. Aspirin use was also associated with a 9.1 and 8.2% reduction in advanced PC risk and the risk of PC with Gleason score ≥7, respectively. Particularly, long-term intake of NSAIDs (≥5 years rather than ≥4 years) was associated with an 11.8% reduction in total PC incidence, while it was the daily dose of aspirin (≥1 pill/day) not the long-term aspirin intake (≥4 years or ≥5 years) that was associated with reduced total PC incidence.

Inconsistent with previous meta-analyses, this meta-analysis found a protective effect of any NSAIDs intake on total PC risk. A recent meta-analysis suggested a positive relation between the intake of any NSAIDs and the risk of PC (20), while neither adverse nor beneficial effects were found by another meta-analysis around the same time (22). To explore the reasons, we made an intensive study of the newly included studies. Although one study illustrated a significantly elevated risk of total PC among any NSAIDs users (18), other studies seem to carry more weight by avoiding detection bias to some extent, due to participants undergoing biopsies independent of PSA levels (51). In addition, similar inconsistency was found between long-term aspirin intake (≥4 or ≥5 years) and the risk of total PC. Based on eight included studies, Liu et al. recently found a significantly reduced PC incidence among those who took aspirin for at least 4 years (22). However, after including 15 studies with more information about the dose and frequency of aspirin intake, this meta-analysis did not find significantly beneficial effects of long-term aspirin intake on the risk of total PC. When it comes to the pooled effects of non-aspirin NSAIDs on the risk of PC, our findings were not completely in accordance with that of previous studies. In this meta-analysis, a non-significant decreased PC risk was detected in “high quality studies,” while an adverse effect of non-aspirin NSAIDs on total PC was observed in “poor quality studies.” It seems that the possibility of a non-significant decreased PC risk for non-aspirin NSAIDs is higher.

Compared to that in all aspirin users (7.0%), our finding of a larger (12.5%) PC risk reduction among those who took at least 1 aspirin tablet daily may indicate a dose related association between the intake of aspirin and the risk of total PC, although we could not pool the data to investigate the association between aspirin intake of less than 1 pill/day and the risk of PC. The above dose related association was reported in several studies (21, 31, 33, 35, 53). However, pooled effect estimates have never generated before. The 12.5% risk reduction observed in this study is in line with the results of recent epidemiological studies. For example, one study conducted by Skriver reported that aspirin intake (≥1 pill/day) was associated with an 11% reduction in PC risk (21). Similarly, Smith et al. suggested that daily aspirin use (≥1 pill/day) also decreased the risk of advanced PC in the NCI-Maryland Prostate Cancer Case-Control Study (31). Experimental studies have also clearly demonstrated that aspirin can inhibit the growth of prostate epithelial cells at the concentration of 0.5 mmol/L, which is the therapeutically relevant concentration (69). In particular, the dose related association between the intake of aspirin and the risk of total PC may result from the fact that lower doses of aspirin may only inhibit the COX-1 isoform, whereas at higher doses, aspirin also inhibits COX-2. Therefore, the dose effect may be explained by the pharmacology of aspirin, which is different from that of other NSAIDs, where there are non-specific inhibition of both COX-1 and COX-2 at all doses. However, with respect to the correlation between the dose of NSAID and PC risk, the pooled effect estimates were not generated due to the limited number of studies; one study suggested that systematic differences may result in a non-dose-dependent association (33). In our opinion, further study is required to obtain convincing evidence.

Regarding long-term intake of NSAIDs or aspirin, the association became less consistent. We found that long-term intake of NSAIDs (≥5 years rather than ≥4 years) and non-aspirin NSAIDs (≥4 or ≥5 years) was associated with reduced PC incidence. The results for NSAIDs or non-aspirin NSAIDs may be valid due to little evidence of heterogeneity. However, no significantly beneficial effects were found between long-term intake of aspirin (≥4 or ≥5 years) and the risk of total PC, though the pooled RRs were <1. Previously, a modest 12–18% reduction in total PC risk was reported among long-term aspirin users (≥4 years) (22, 70). Moreover, Ma et al. recently found that long-term use of aspirin (≥5 years) or non-aspirin NSAIDs (≥3 years) decreased the risk of PC (49). It is interesting to consider what accounts for the final pooled effects of long-term aspirin use. First, there is a higher likelihood that those who take aspirin for a long time simultaneously take other medications for a long time, which may greatly impact the perceived effects of long-term aspirin use. Second, considering the substantial heterogeneity among the included studies, the crudely estimated duration of aspirin intake in some studies may have led to over- or underestimation of the real effects of long-term aspirin use on PC incidence.

As for the geographic difference, we observed that the intake of NSAIDs or aspirin was associated with a decreased PC risk in both Europe and North America, though there was no statistical difference in PC risk due to the intake of NSAIDs in Europe. Additionally, a negative association was also observed between PC incidence and the intake of non-aspirin NSAIDs in North America, while there was an insignificant positive relation between PC incidence and the intake of non-aspirin NSAIDs in Europe. Some previous studies indicated that the effect of the intake of NSAIDs on PC incidence seemed to vary by geographic region, which may result from a potential bias. For example, studies of European men reported that the use of NSAIDs was associated with an increased risk of total PC (33, 17, 44), while a reduced risk of total PC was found among NSAIDs users from North America (40, 43, 47, 71). Given that previously, PSA testing was practiced less frequently in Europe than in North America, PC in European men taking NSAIDs were likely to be missed and to be detected at a later stage, which may account for the positive associations to some degree. However, recent studies from Europe showed an overall modest protective effect of the intake of NSAIDs or non-aspirin NSAIDs on the incidence of PC, which could be attributed to the increasing popularity of PSA screening (32, 49, 50).

Furthermore, we detected that there was no remaining association between the intake of NSAIDs and PC incidence when we restricted our analysis to studies adjusting for comorbidities. The final pooled effects of aspirin intake seemed to be influenced by the concomitant use of other medications. Comorbidity and the concomitant use of other medications could influence the risk of PC and introduce an indication bias due to the fact that several comorbidities (cardiovascular and rheumatologic diseases) were the main reasons for their intake of NSAIDs. In addition, cardiovascular events and PC shared several common risk factors, such as smoking, alcohol, obesity, and low levels of physical activity. Thus, PC may be more prevalent in those with certain risk factors than in the general population (50). It should also be noted that other medications used simultaneously, such as statins and metformin, were commonly prescribed to NSAIDs users and their combined effects on the risk of PC should not be neglected. Interestingly, statins also showed some promising chemopreventive effects against PC, although insufficient evidence from multiple reports was merely suggestive rather than conclusive. For example, Ma et al. found that the protective effect of aspirin was less pronounced among those who took aspirin and statins simultaneously (49).

Meanwhile, we found that the intake of aspirin, rather than NSAIDs or non-aspirin NSAIDs, was associated with a greater decrease in the risk of advanced PC and PC with Gleason score ≥7, with little heterogeneity (*I*^2^ = 16.20 and 28.40%, respectively). Although the final pooled effect of aspirin intake on the incidence of advanced PC was consistent with the results of previous studies, the degree of risk reduction in those studies varied from 11 to 30% (20, 22, 70, 72). For fear of misclassification of the stage due to the lack of complete information based on TNM classifications, a pooled analysis was further performed to investigate the association between drug use and PC with Gleason score ≥7. However, this association was not consistent either (21, 53, 54, 66).

Considering the different effect estimates included, subgroup analyses were conducted based on different effect estimates. Interestingly, the subgroup analysis on ORs, instead of RRs or HRs, demonstrated a negative trend of PC risk in NSAIDs or aspirin users. There seems to be further room for methodological improvement in these studies.

### Limitations

The limitations of this study should also be acknowledged. Firstly, heterogeneity was an inevitable problem, and it was also a very significant problem in the previous meta-analyses. Though sensitivity analyses indicated that none of the individual datasets substantially altered the pooled effect estimates of drugs intake on PC incidence, the summarized estimates in this meta-analysis may be ambiguous to the public and should be treated with caution due to a considerable heterogeneity. In our meta-analysis, methodological heterogeneities across included studies were observed and could not be reduced and interpreted in some subgroup analyses. Specifically, the definitions of drug intake, ages of participants, and sample sizes of studies were rather different among included studies, for example, the definitions of drug intake vary from daily to once a month, making it difficult to determine an optimum dividing-class value to conduct corresponding stratified analyses and resulting in a certain level of heterogeneity undoubtedly. Accordingly, the reference groups were defined differently, which may also bias the pooled effect estimates. Moreover, it may be inappropriate to choose a single effect estimate to pool the data, though the heterogeneity across included studies could not explained completely by stratified analyses based on study design or types of effect measures.

Secondly, possible publication bias remains another potential impact, although we included as many English and Chinese databases as possible, and no publication bias was detected according to the Begg's or Egger's tests. However, studies with negative results are less likely to be published in indexed journals. Thus, those unpublished negative studies may result in possible publication bias, though those published in “gray literature,” such as theses, book chapters, and meeting abstracts, were also searched particularly in this meta-analysis. Furthermore, another possible publication bias could attribute to the exclusion of studies without available information, which may also lead to the downgrading of evidence.

Thirdly, the inherent limitations resulting from the design of included studies might involve a certain level of recall bias and selection bias, which may contribute to potential misclassification of exposure and outcome. Evaluations of drug use based on interviews or questionnaires are likely to be prone to recall related measurement errors, particularly regarding the dose and duration of drug intake (49). Although the assessment of the intake of NSAIDs obtained by complete prescription histories minimize the risk of misclassification as much as possible, some NSAIDs may be bought over-the-counter, and therefore, complete and accurate information was not available. In addition, it also should be mentioned that the over-the-counter use of NSAIDs was not included in some studies. Furthermore, there was likely drop-in and drop-out of NSAIDs users if the updated data was insufficient (51).

Moreover, the underestimation of PC incidence resulting from the potential ability of NSAIDs to alter PSA levels may lead to another type of selection bias. PSA levels were reported to be lowered by a modest 6% in individuals who used NSAIDs for more than 5 years (73). As is well known, PSA screening plays a leading role in PC detection. Therefore, some studies may have suffered from a selection bias, in which only men with abnormal PSA levels were referred for biopsy. In other words, men with lower PSA levels due to the use of NSAIDs may have received fewer biopsies, and therefore, their PC was detected at a later stage (31, 51).

Finally, studies of men with advanced PC whose PSA levels were much higher than those of men with early-stage disease would not suffer from such a detection bias because their PSA levels at diagnosis would still meet the biopsy criteria even though their use of NSAIDs may result in a modest reduction in PSA levels (31). Thus, the relationship between the intake of NSAIDs and advanced PC is unlikely to be influenced by disease detection bias caused by the use of NSAIDs. However, it was also a concern that the protective effect of the intake of NSAIDs on advanced PC may be over-estimated due to the more frequent PSA screening of patients with advanced PC, thereby leading to earlier cancer detection (19), which could be a source of screening bias.

## Conclusions

The results of this meta-analysis provided quantitative evidence regarding the protective effect of the intake of any type of NSAIDs on the risk of PC, especially among those with long-term NSAID use (≥5 years). Moreover, aspirin intake was also associated with a decreased risk of PC, and it was the daily dose (≥1 pill/day) not the long-term intake of aspirin (≥4 or ≥5 years) that was associated with the reduced incidence of PC. No significantly adverse or beneficial effects were found between the intake of non-aspirin NSAIDs and the risk of total PC, advanced PC, or PC with Gleason score ≥7. It is necessary to perform further well-designed large-scale randomized controlled trials to draw a definitive conclusion, which could adjust for the potential known and unknown confounders.

## Author contributions

ZS and TO conceived and designed the study. ZS, XW, KY, JW, HY, and QW contributed to the extraction of data and analysis of the results. All the authors contributed in writing and editing the manuscript.

### Conflict of interest statement

The authors declare that the research was conducted in the absence of any commercial or financial relationships that could be construed as a potential conflict of interest.
