# Supplementary material for: Intake of Non-steroidal Anti-inflammatory Drugs and the Risk of Prostate Cancer: A Meta-Analysis
Source: Front Oncol. 2018 Oct 23;8:437. doi: 10.3389/fonc.2018.00437 (PMC6206266; doi:10.3389/fonc.2018.00437)
Supplement: Supplementary file 1 [file Table_1.DOC]

**Supplementary File S1**

**S1 Newcastle-Ottawa Scale adapted for cross-sectional studies**

**Selection:** (Maximum 5 stars)

1) Representativeness of the sample:

a) Truly representative of the average in the target population. * (all subjects or random sampling)

b) Somewhat representative of the average in the target population. * (non-random sampling)

c) Selected group of users.

d) No description of the sampling strategy.

2) Sample size:

a) Justified and satisfactory. *

b) Not justified.

3) Non-respondents:

a) Comparability between respondents and non-respondents characteristics is established, and the response rate is satisfactory. *

b) The response rate is unsatisfactory, or the comparability between respondents and non-respondents is unsatisfactory.

c) No description of the response rate or the characteristics of the responders and the non-responders.

4) Ascertainment of the exposure (risk factor):

a) Validated measurement tool. **

b) Non-validated measurement tool, but the tool is available or described.*

c) No description of the measurement tool.

**Comparability:** (Maximum 2 stars)

1) The subjects in different outcome groups are comparable, based on the study design or analysis. Confounding factors are controlled.

a) The study controls for the most important factor (select one). *

b) The study control for any additional factor. *

**Outcome:** (Maximum 3 stars)

1) Assessment of the outcome:

a) Independent blind assessment. **

b) Record linkage. **

c) Self report. *

d) No description.

2) Statistical test:

a) The statistical test used to analyze the data is clearly described and appropriate, and the measurement of the association is presented, including confidence intervals and the probability level (p value). *

b) The statistical test is not appropriate, not described or incomplete.

Supplementary File S2

**S2 NOS scores of included studies**

| **Study** | **Published year** | **Study design** | **Types of Drug** | **NOS** | | | |
| --- | --- | --- | --- | --- | --- | --- | --- |
|  |  |  |  | **Selection** | **Comparability** | **Exposure or outcome** | **Total score** |
| Murtola (49) | 2017 | Cohort | NSAIDs | *** | * | *** | 7 |
| Smith (31) | 2017 | Case-Control | Aspirin | **** | ** | * | 7 |
| Doat (32) | 2017 | Case-Control | NSAIDs, Aspirin, non-aspirin NSAIDs | **** | * | ** | 7 |
| Ma (50) | 2017 | Cohort | Aspirin,  non-aspirin NSAIDs | *** | * | *** | 7 |
| Kang (18) | 2017 | Cohort | NSAIDs, Aspirin | *** | ** | *** | 8 |
| Downer (68) | 2017 | Cohort | Aspirin | ** | ** | *** | 7 |
| Skriver (21) | 2016 | Case-Control | Aspirin,  non-aspirin NSAIDs | *** | ** | ** | 7 |
| Huang (69) | 2016 | Cross-sectional | Aspirin | *** | ** | ** | 7 |
| Lapi (51) | 2016 | Cohort | Aspirin | *** | ** | ** | 7 |
| Cao (23) | 2016 | Cohort | Aspirin | *** | ** | *** | 8 |
| Vidal (52) | 2015 | Cohort | NSAIDs | ** | ** | ** | 6 |
| Nordström (53) | 2015 | Cohort | Aspirin | *** | ** | ** | 7 |
| Veitonmaki (33) | 2013 | Case-Control | Aspirin, NSAIDs | **** | ** | ** | 8 |
| Shebl (54) | 2012 | Cohort | Aspirin,  non-aspirin NSAIDs | *** | ** | ** | 7 |
| Dhillon (55) | 2011 | Cohort | Aspirin | ** | ** | *** | 7 |
| Murad (34) | 2011 | Case-Control | NSAIDs, Aspirin, non-aspirin NSAIDs | *** | ** | ** | 7 |
| Mahmud (35) | 2011 | Case-Control | NSAIDs, Aspirin, non-aspirin NSAIDs | **** | ** | * | 7 |
| Brasky (56) | 2010 | Cohort | Aspirin,  non-aspirin NSAIDs | *** | ** | ** | 7 |
| Salinas (36) | 2010 | Case-Control | Aspirin,  non-aspirin NSAIDs | **** | * | ** | 7 |
| Coogan (37) | 2010 | Case-Control | NSAIDs | ** | ** | ** | 6 |
| Siemes (57) | 2008 | Cohort | NSAIDs, Aspirin, non-aspirin NSAIDs | *** | * | *** | 7 |
| Jacobs (58) | 2007 | Cohort | Aspirin | *** | ** | *** | 8 |
| Harris (38) | 2007 | Case-Control | Aspirin | *** | * | ** | 6 |
| Mahmud (70) | 2006 | Cross-sectional | NSAIDs | **** | ** | * | 7 |
| Menezes (39) | 2006 | Case-Control | Aspirin | *** | * | ** | 6 |
| Bosetti (40) | 2006 | Case-Control | Aspirin | *** | * | ** | 6 |
| Dasgupta (48) | 2006 | Case-Control | NSAIDs, Aspirin | *** | ** | * | 6 |
| Jacobs (60) | 2005 | Cohort | NSAIDs | *** | ** | *** | 8 |
| Platz (59) | 2005 | Cohort | NSAIDs, Aspirin, non-aspirin NSAIDs | *** | ** | *** | 8 |
| Rodríguez (41) | 2004 | Case-Control | Aspirin, non-aspirin NSAIDs | *** | * | ** | 6 |
| Perron (42) | 2003 | Case-Control | NSAIDs, Aspirin | ** | ** | ** | 6 |
| Friis (62) | 2003 | Cohort | Aspirin | *** | * | *** | 7 |
| Sørensen (61) | 2003 | Cohort | Non-aspirin NSAIDs | ** |  | *** | 6 |
| Leitzmann (67) | 2002 | Cohort | Aspirin | ** | ** | ** | 6 |
| Habel (64) | 2002 | Cohort | Aspirin | *** | * | *** | 7 |
| Roberts (63) | 2002 | Cohort | NSAIDs | *** |  | ** | 5 |
| Irani (43) | 2002 | Case-Control | NSAIDs, Aspirin, non-aspirin NSAIDs | **** | ** | ** | 8 |
| Langman (45) | 2000 | Case-Control | NSAIDs | **** | * | ** | 7 |
| Nelson (44) | 2000 | Case-Control | NSAIDs | *** | * | ** | 6 |
| Norrish (46) | 1998 | Case-Control | NSAIDs, Aspirin, non-aspirin NSAIDs | *** | ** | ** | 6 |
| Neugut (47) | 1998 | Case-Control | Aspirin | *** | ** | ** | 7 |
| Schreinemache (65)rs | 1994 | Cohort | Aspirin | *** | ** | ** | 7 |
| Paganini-Hill (66) | 1989 | Cohort | Aspirin | ** | ** | ** | 6 |
